# Editorial: Biotrophic Plant-Microbe Interactions

**DOI:** 10.3389/fpls.2017.00192

**Published:** 2017-02-13

**Authors:** Pietro D. Spanu, Ralph Panstruga

**Affiliations:** ^1^Department of Life Sciences, Imperial College LondonLondon, UK; ^2^Unit of Plant Molecular Cell Biology, Institute for Biology I, RWTH Aachen UniversityAachen, Germany

**Keywords:** obligate biotrophs, hemibiotrophs, necrotrophs, endophytes, symbionts, haustorium, saprotrophs, mycorrhiza

## Biotrophs and other partners

Organisms inhabit the biosphere not as isolated entities: they interact with others. These may be individuals of the same species. In fact, the most common interactions are likely to be with very different beings. The interactions may be fleeting, or life-long, they may be simply sharing the same space, or may be complex behavioral and developmental processes (Buxa et al.; Genre and Russo) from which one or both partners derive an advantage and improve their reproductive success.

## Interactions defined by exchange of food

Plants are no exception to this universal rule: they share their personal space with myriads of microbes (Souza et al.). In the case of living plants, this may result in seemingly neutral (Shaw et al.; Voisey et al.), mutually beneficial (Banhara et al.; Calabrese et al.; Manck-Gotzenberger and Requena) or detrimental (Bindschedler et al.; Langenbach et al.) interactions; the respective microbes are commonly called endophytes, symbionts and pathogens, respectively. The best studied interactions are those which result in transfer of resources, such as nutrients, from one partner to the other. These “trophic” relations are frequently used to categorize interactions between plants and microbes. In simple terms, when the plants remain alive during the nutrient exchanges, we talk of “biotrophic interactions” and refer to the microbes as “biotrophs” (Spanu and Kämper, 2010). This is typically the case in symbiotic relationships, but also in some instances of parasitism. Biotrophy is thus contrasted to “necrotrophy,” that is when the microbes kill plant cells and tissues, to feed off the remains, which is characteristic for several phytopathogens (Shaw et al.). In practice, we recognize many intermediate states characterized by temporal and/or spatial transitions between biotrophy and necrotrophy, and refer to these relations as hemibiotrophic (Vleeshouwers and Oliver, 2014). When microbes are simply able to feed off dead plant remains whilst playing no part in the killing, we call them saprotrophs (Lewis, 1973).

The consensus is that saprotrophy is the ancestral status for plant-associated microbes (Martin et al., 2016). Requirements needed to access nutrients from dead plants include the ability to degrade biopolymers, actively explore solid matter, and deal with potentially toxic compounds left by the dead plant. Interacting with a live plant partner requires much more complex and sophisticated mechanisms, first and foremost the capacity to deal with and take control of plant immunity (Ruhe et al.), which evolved to protect plants from unwanted, harmful encroachment. The ability to manipulate host metabolism and to redirect nutrients for their own benefit are further essential skills for these types of microbes (Calabrese et al.; Manck-Gotzenberger and Requena). To realize these necessities, many microbial species evolved secreted effector proteins that exert various activities in the plant host (Kunjeti et al.; Petre et al.; Pitino et al.; Xiang et al.).

## Is killing simpler than sharing?

For many years, biotrophy has been regarded as the most complex form of trophic relation between organisms. This has led many to consider biotrophy to be more “advanced” (Lewis, 1973)—perhaps a controversial and not particularly useful term. In recent years, there has been a revision of this: true necrotroph lifestyles are supported by highly sophisticated/evolved killing mechanisms (Oliver and Solomon, 2010). They are not simple blunderers that happen to have developed from saprotrophic organisms (Delaye et al., 2013).

It has been widely accepted that the distinction between biotrophic and necrotrophic interactions may also be evident in distinct pathways that host plants use to signal responses to the invading microbe. Thus, salicylic acid-mediated responses are regarded as typical of reactions to biotrophic attack, while jasmonic acid- and ethylene-mediated ones are believed to be associated with necrotrophy (Glazebrook, 2005). This distinction is now brought into question, with data revealing roles for jasmonic acid signaling in the unquestionably biotrophic interaction of grapevine with downy mildew (Guerreiro et al.).

## The compulsion to feed off life: obligate biotrophs

This revision notwithstanding, biotrophic microbes have developed exquisitely complex mechanisms to access plant resources. The rich niche represented by a plant host is characterized by having fewer microbial competitors than, say, soil or water. So, unlocking access confers a significant advantage: abundant resources available with “predictable” frequency throughout time and space. Once this space was occupied, some microbes appear to have lost the original capacity to grow on non-live material: these are recognized as the “obligate biotrophs.” The most extreme of the obligate biotrophs have become so dependent of a live host that we are unable to recreate a suitable environment in axenic cultures under laboratory conditions. Examples of these are the very ancient mutualistic symbiont arbuscular mycorrhizal fungi (Buxa et al.; Genre and Russo) that are near ubiquitous colonizers of plant roots, the common powdery mildew (Gafni et al.; Xu et al.; Bindschedler et al.; Bourras et al.; Orton and Brown; Rajaraman et al.; Zheng et al.) and rust (Tang et al.; Langenbach et al.; Liu et al.; Liu et al.; Petre et al.) fungi (from taxonomically very distant groups, namely ascomycetes and basidiomycetes), as well as some of the oomycetes such as downy mildews (Guerreiro et al.; Kulkarni et al.; Raaymakers and van den Ackerveken) and white rusts (Ruhe et al.).

It is important to remember that some organisms are likely to be actually obligate biotrophs in nature, even if they are still culturable in axenic conditions in the laboratory. The fungi that cause smuts on several plant hosts, the Ustilaginaceae, are thus naturally obligate biotrophs, in the sense that there is no record of growth and reproduction in non-plant or soil environments, in the wild (Brefort et al., 2009).

## One haustorium does not make a biotroph (pace aristotle)

In addition to complex molecular mechanisms aimed at tuning plant immunity, many biotrophic microbial eukaryotes produce complicated morphological structures exquisitely adapted at abstracting nutrient from plant cells: these are termed haustoria. They are terminal branch extensions of the microbial cells and hyphae that penetrate through the cell walls. The most elaborate of these are observed in the arbuscular mycorrhizae, which produce the eponymous “arbuscules” resembling small trees or bushes (hence the name; Calabrese et al.; Manck-Gotzenberger and Requena). Similar structures are made by some of the powdery mildews, in a marvelous example of the evolutionary convergence principle (Parniske, 2000). At the other end of the complexity spectrum, we find the simple bulbous haustoria made by rust fungi and oomycetes. A common feature of all true haustoria/arbuscules is that they are formed by hyphae that penetrate the host cell wall, but do not perforate the plant cell membrane. Rather, the plasma membrane invaginates and gives rise to a new structure, the perihaustorial/periarbuscular membrane, with very special properties that are distinct from the contiguous plasma membrane (Koh et al., 2005). In the organisms that make them, most of the crucial nutrient and signaling exchanges are thought to happen here (Voegele and Mendgen, 2003).

However, biotrophs are not restricted to haustoria-forming fungi. There are plenty of purely apoplastic biotrophs, i.e. biotrophs that do not establish any highly specialized haustoria. Examples of this comprise the fungal tomato pathogen *Cladosporium fulvum* (Joosten and de Wit, 1999) and the corn smut pathogen *U. maydis* (Brefort et al., 2009). Self-evidently, exchanges between plant host and the microbial “guest” must take place in the apoplast in these instances. It should be noted, though, that apoplastic signaling can also be relevant in interactions where haustoria are formed (Raaymakers and van den Ackerveken). A most extreme form of apoplastic biotrophy is evident in the so-called “endophytic” microbes (Voisey et al.). These are microorganisms that colonize plant hosts, *prima facie* asymptomatically. In recent years, the importance and potential of these interactions has been recognized and led to concerted efforts at exploiting the advantages conferred on the host in terms of enhanced resistance to pathogen infection, for example (Johnson et al., 2013). Conversely, there are also pathogens such as many of the *Phytophthora* species that are traditionally regarded as necrotrophs (at least for the most agronomically significant part of their infection cycle) that make *bona fide* haustoria (Whisson et al., 2016).

## Hemibiotrophs: interactions that straddle the divide

Typical hemibiotrophic microbes start off with an asymptomatic phase (Di et al.), which then switches to a killing spree—the necrotrophic phase when host cell death is commonly associated with extensive microbial colonization and sporulation. An intriguing question is whether the asymptomatic phase can be equated with true biotrophy. The crucial point is whether at this time the microbe is active, growing and taking up nutrients from the host (in which case we have true biotrophy), or whether they are simply surviving on endogenous stored reserves (in which case they are not really biotrophs). A further possibility is that the microbial partner is actually dormant and hence it might be truly justified to call this a latent phase. Of course, a last option is that the microbe is simply undetectable, relative to the clearly visible biomass at later stages, when exponential growth accompanies the necrotrophic phase, and sporulation. Defining which of these is true is challenging because there is very little microbial biomass per plant tissue at this time. Molecular biology-based methodologies or advanced transcriptome analysis are now sensitive enough (O'Connell et al., 2012; Bindschedler et al.; Kulkarni et al.; Kunjeti et al.; Shu et al.), but biochemical and physiological analysis may be difficult, or impossible, with current methodologies.

If the first phase of infection in hemibiotrophs is truly biotrophic, we may then ask ourselves what the position of archetypal necrotrophs really is. In *Botrytis*, that phase is usually described as latent. But is it? It is becoming apparent that there are intriguing instances of truly endophytic *Botrytis* species (Shaw et al.). These are normally concealed due to their intrinsically asymptomatic nature. Then there are pathogens that do not know what they are: take *Leptosphaeria maculans*, the fungus that causes black-leg on brassicas (Sonah et al.). These start off with a short a symptomatic/biotrophic infection on leaves, which switch to necrotrophy visible as dead leaf lesions. The disease then turns to an asymptomatic/biotrophic and endophytic stage in which the fungus grows intercellularly, reaching the crown of the mature plant where necrotrophic cankers are formed. *L. maculans* is clearly a fungus with many tricks up its sleeve.

## The technical challenges of studying biotrophy

A significant number of microbes that grow on plants causing disease, or even those with a mutualistic steady state, cannot be grown in axenic (“pure”) culture. This big drawback severely limits experimentation, as it is difficult to collect enough biological material for biochemical and physiological experimentation. All manipulations are to be done in presence of a host, complicating biochemical and other types of analyses. Additionally, with few exceptions, genetic manipulations of these microorganisms are either extremely laborious or impossible at present. This hampers tremendously cell biological and functional analysis of the respective plant-microbe interactions (Bindschedler et al.). Novel techniques and methodologies, e.g., for the visualization of encounters between plants and biotrophs (Ghareeb et al.) are thus highly desired to further expand the tool-box to study these organisms.

## Resistance against biotrophic pathogens

The plant immune system evolved to cope also with biotrophic pathogens. A key initial event of immunity is the perception of pathogen-derived molecules (“patterns”) by membrane-resident receptors (often dubbed pattern recognition receptors; Raaymakers and van den Ackerveken; Rajaraman et al.). A second layer of plant defense rests on the direct or indirect recognition of secreted pathogen effectors (“avirulence proteins”; Bourras et al.) by typically cytoplasmic immune sensors (“resistance proteins”; also termed nucleotide binding-oligomerisation domain (NOD)-like receptors) that usually confer isolate-specific resistance (Williams et al.). Execution of the actual defense response often involves re-organization of the host cytoskeleton (Tang et al.) and secretory activity (Xu et al.; Liu et al.). In addition, phytohormone signaling (Di et al.; Guerreiro et al.) and other plant components may contribute to resistance (Liu et al.), or immunity might be conditioned by the absence of essential host factors (Zheng et al.).

## Mutual influence of biotrophs and other microbes

A largely neglected aspect of the biology of interactions between plants and biotrophic microbes is their modulation by any third partner(s). In fact, the rhizosphere and phyllosphere of plants is colonized by various epi-/endophytes, and multiple pathogens and/or symbionts may occur at the same time on a given plant. Thus, biotrophic microbes may need to compete with other microorganisms for their ecological niche (Ruhe et al.). This might cause altered infection phenotypes of biotrophic pathogens in the presence of other pathogens (Orton and Brown) or epi-/endophytes (Gafni et al.) and also could result in modulation of symbiotic interactions by phytopathogens (Souza et al.).

## Concluding remarks

Despite significant progress in various areas, the analysis of interactions between plants and biotrophic microbes remains a challenging business. In the short term, we expect that expanding research efforts in those areas such as gen- and other –omics is likely to yield dividends even for the more intractable associations (Bindschedler et al.). Moreover, we predict that a mechanistic understanding of how the plethora of effectors, which appear to be encoded by all microbes interacting with plants, will undoubtedly progress our knowledge of the complexities of interkingdom signaling. It remains to be seen how all of this may eventually be translated into a capacity to intervene to mitigate the action of harmful pathogens and further the activity of desirable ones.

## Author contributions

RP and PS jointly wrote and edited the text.

## Funding

PS was supported by the BBSRC grant BB/M000710/1. Research in the lab of RP is currently supported by the following grants of the Deutsche Forschungsgemeinschaft (DFG): ERA-CAPS “DURESTrit” (PA861/13-1), priority program SPP1819 “Rapid evolutionary adaptation: potential and constraints; PA861/14-1) and the ANR-DFG cooperation “X-KINGDOM-MIF” (PA861/15-1).

### Conflict of interest statement

The authors declare that the research was conducted in the absence of any commercial or financial relationships that could be construed as a potential conflict of interest.
